# Predictors of Mental Health among the General Population of U.S. Adults Eight Months into the COVID-19 Pandemic

**DOI:** 10.4236/psych.2022.133029

**Published:** 2022-03-30

**Authors:** Elizabeth Flanagan Balawajder, Bruce G. Taylor, Phoebe A. Lamuda, Kai MacLean, Harold A. Pollack, John A. Schneider

**Affiliations:** 1 Public Health Department, NORC at the University of Chicago, Chicago, USA; 2 Crown Family School of Social Work, Policy and Practice, University of Chicago, Chicago, USA; 3 Department of Medicine and Public Health Sciences, University of Chicago, Chicago, USA

**Keywords:** COVID-19, Mental Health, MHI-5

## Abstract

**Background:**

The COVID-19 pandemic has had profound impacts on mental health. We examined whether mental health differed based on sociodemographic and background characteristics, political party affiliation, and concerns about COVID-19.

**Methods:**

A cross-sectional, national sample of 1095 U.S. adults were surveyed October 22–26, 2020. The survey collected information on demographics, risk and protective behaviors for COVID-19, and mental health using the Mental Health Inventory-5 (MHI-5) scale. Independent samples t-tests, one-way Analysis of Variance tests, and a multivariable linear regression model were conducted.

**Results:**

Regression results showed respondents with criminal justice (B = −6.56, 95% CI = −10.05, −3.06) or opioid misuse (B = −9.98, 95% CI = −14.74, −5.23) histories reported poorer mental health than those without. Those who took protective behaviors (e.g., wearing masks) reported poorer mental health compared to those who indicated protective behaviors were unnecessary (B = 7.00, 95% CI = 1.61, 12.38) while those who took at least one risk behavior (e.g., eating in a restaurant) reported better mental health than those who did not.

**Conclusions:**

Our study shows that certain groups have experienced poorer mental health during the COVID-19 pandemic, suggesting that mental health should continue to be monitored so that public health interventions and messaging help prevent the spread of COVID-19 without increasing poor mental health outcomes.

## 1. Background and Introduction

On March 11, 2020, COVID-19 was declared a global pandemic by the World Health Organization. Approximately 19 months after being declared a pandemic, there have been over 240 million confirmed cases and almost five million deaths worldwide, including 45 million cases and over 700,000 deaths in the United States (Johns Hopkins University & Medicine, 2021). The virus and the social distancing policies that were implemented to mitigate its spread have had far-reaching implications on society, spurring discussion of the mental health implications since the earliest days of the pandemic (Cullen et al., 2020; Cypress, 2021; Pfefferbaum & North, 2020).

Research has demonstrated that adults across the world have been experiencing poor mental health outcomes, such as high levels of anxiety and depression since the pandemic began (Usher et al., 2020; Vindegaard & Benros, 2020; Vahratian et al., 2021). For example, a study in February 2020 with the general public in China found that over half of respondents reported a moderate to severe psychological impact, including 29% with anxiety and 17% with depression symptoms (Wang et al., 2020). Studies examining mental health in the United States starting in March 2020 have had similar findings. In nationally representative surveys collected in June 2020 and September 2020, over 40% of adults reported experiencing at least one adverse mental health condition (Czeisler et al., 2021; Czeisler et al., 2020). A longitudinal study measuring depression between February 2019 and July 2020 found that depression rates were approximately 50% higher than before the pandemic (Giuntella et al., 2021). Of further concern, one study found that many who experience these symptoms do not seek mental health treatment or services (Vahratian et al., 2021).

Literature drawing on disaster mental health principles suggests that the pandemic’s mental health implications are just beginning since the mental health impacts of other disasters have lasted 3 – 5 years post-disaster (Alkhayyat & Pankhania, 2020; North et al., 2021; Pfefferbaum & North, 2020) and some literature even suggests that mental health impacts of the pandemic will last several years longer than the impacts on physical health and healthcare systems (Kohli & Virani Salim, 2020).

COVID-19 affects people differently based on sociocontextual factors and a person’s characteristics and beliefs (Bernabe-Valero et al., 2021). Therefore, while poor mental health outcomes have been observed across the general adult population, some groups have been more affected than others by the pandemic. For example, those with pre-existing mental health conditions or health conditions that put people at greater risk for severe COVID-19 have exhibited more distress, anxiety, and depression (Holman et al., 2020; Veldhuis et al., 2021; Zhou et al., 2020). Additionally, younger adults, sexual minorities, essential workers, and caregivers may be disproportionately affected by poor mental health during the pandemic (Holland et al., 2021; Czeisler et al., 2020; Zhou et al., 2020). Similarly, multiple studies have shown that those who have been personally affected by COVID-19, through a diagnosis or death of someone they knew, as well as those who lost their employment or wages, have more adverse mental health symptoms (Czeisler et al., 2021; Holland et al., 2021; Czeisler et al., 2020). While findings from these studies might suggest that concerns related to contracting or adverse health outcomes from COVID-19 affect mental health, research has not yet examined how this concept is expressed through either risk or preventive behavior taking in the United States. Results have been mixed on differences between racial groups (Czeisler et al., 2020; Veldhuis et al., 2021; Zhou et al., 2020).

Prior to the COVID-19 pandemic, the United States public health system was working to combat the opioid epidemic, with nearly 50,000 overdose deaths in 2019 (Mattson, 2021). There has been some discussion on the pandemic’s adverse impact on those with an opioid use disorder, such as limited access to treatment as healthcare facilities focus efforts on addressing COVID-19 (Schimmel & Manini, 2020; Khatri & Perrone, 2020) and the literature has also mentioned that exacerbated mental health conditions during COVID-19 may trigger opioid use (Khatri & Perrone, 2020). However, data has not yet focused on the mental health of those with a history of opioid misuse. Many people who have misused opioids also have been involved in the criminal justice system (Winkelman et al., 2018), which is another factor that may make individuals particularly vulnerable to the effects of the pandemic. Some research suggests that COVID-19 has exacerbated challenges often faced by those with criminal justice involvement histories such as unstable housing, but there has been little focus on this population (Ramaswamy et al., 2020).

This study explores the mental health of a national sample and identifies groups who may be particularly at risk for poorer mental health outcomes during the pandemic, including those based on age, race, political party affiliation, opioid misuse and criminal justice histories. Our data augments the literature on mental health disparities between different sociodemographic and COVID-affected groups and also highlights differences in mental health based on engagement in COVID-19 risk and protective behaviors. This data uniquely captures the mental health symptoms of a national sample in October 2020, approximately eight months following the start of the pandemic in the U.S., five months following the initiation of the 2020 racism protests, just one week prior to the 2020 presidential election and two weeks prior to the first announcement of an effective vaccine. Thus, this cross-sectional study sheds light on key aspects of the mental health of the nation at a pivotal time point since the start of the pandemic and assesses which groups may be particularly susceptible to poor mental health outcomes.

## 2. Methods

The findings reported in this study were drawn from a cross-sectional survey collected between October 22, 2020 and October 26, 2020 from a national sample of U.S. adults. Institutional Review Board (IRB) approval for the conducting of human subjects research was obtained from the lead author’s organization.

### Sample

Participants were chosen for the study to provide a representative snapshot of the mental health of adults across the U.S. The research team drew a cross-sectional random sample of participants (n = 1095 adults 18+, with 5% missing data n = 1040) using the AmeriSpeak^®^ probability-based panel. The AmeriSpeak^®^ panel consists of over 35,000 households, designed to be representative of the U.S. household population. The AmeriSpeak^®^ panel is built from a stratified random sample of U.S. households selected and sampled using area probability and address-based sampling, with a known, nonzero probability of selection from the NORC at the University of Chicago (NORC) National Sample Frame. Sampled households are contacted by multiple modalities to capture harder-to-reach participants. Sample coverage for the panel is about 97% of the U.S. household population (Dennis, 2019). With an annual panel retention rate of about 85%, (Dennis, 2019) the AmeriSpeak^®^ sample compares favorably to the U.S. Census American Community Survey; on average the samples are different by under 1.5%, by sex, age group, race/ethnicity, education, marital status, employment, income, region, and home Internet access (Bilgen et al., 2018; Montgomery et al., 2016). AmeriSpeak^®^ has maintained a 37% weighted panel household recruitment rate through the use of the second stage of in-person recruitment for non-responders to capture harder-to-reach populations. Accounting for age, race/ethnicity, education, and sex, the AmeriSpeak^®^ panel implements monthly omnibus surveys using a probability sample of adults.

### Recruitment and Data Collection

To recruit participants for the survey, emails and texts were sent to a randomly selected group of panelists (n = 4358) from the AmeriSpeak^®^ panel. Emails and texted described the study and the first page of the survey included the informed consent. Participants who did not respond to the initial invitation were contacted multiple times by email, text and phone. Participants received a $4 incentive. The survey was offered in English and Spanish, and participants could complete it on a secure web survey or by phone. Of the 4358 contacted, 1095 (25.12%) completed the survey.

### Measures

#### MHI-5.

Based on the Mental Health Inventory (Veit & Ware, 1983) to assess psychological well-being and distress in the general population, the MHI-5 (Berwick et al., 1991) comprises five items from the original 38 item pool and has proven to be valid and reliable for use with different subgroups and in different cultures (Ware et al., 1993). The MHI-5 uses a five-item scale, with participants indicating how often they experienced a certain feeling over the past month using a 6-point Likert-type scale ranging from “All of the time” to “None of the time”. Example scale items include “been a very nervous person” and “felt calm and peaceful”. Two items were reverse coded before computing the scale mean ranging from 1 to 6. This value was transformed to a 0 – 100 scale using a linear transformation, with 100 indicating optimal mental health. The Cronbach’s alpha score for our MHI-5 measures was good (0.861).

#### COVID-19 Protective and Risk Behaviors.

To assess concern for COVID-19, participants were asked to indicate whether they engaged in protective behaviors and risk behaviors over the past two weeks. Protective behaviors included items such as “wearing a mask or face covering when leaving home” and “washing/sanitizing hands more than usual.” Participants could respond in one of three ways: 1) yes, 2) no, I don’t think it’s necessary, and 3) no, I would like to but I cannot. Participants were also asked whether they engaged in activities known to put people at higher risk of contracting COVID in October 2020, such as “attending a gathering with more than 10 people” or being “within six feet of someone outside your household when you were not wearing a mask.” Protective behaviors were categorized into four groups during analysis: 1) taking all four behaviors, 2) taking 2 – 3 protective behaviors and indicating 1 – 2 weren’t necessary, 3) indicating most (at least 3) behaviors weren’t necessary, and 4) unable to take at least one behavior. Risk behavior was dichotomized into taking at least one risk behavior or not taking any risk behaviors.

#### Demographics and background.

Data were collected on the sociodemographic and background characteristics of respondents, including age, biological sex, race, education, employment, political party, and income. These variables were categorized into categories as shown in Table 1. Respondents were also asked whether they had ever misused opioids, been incarcerated, or been convicted of a crime during their lifetime. Incarceration and conviction were combined into a single variable so that positive response for either indicated a personal history of criminal justice involvement.

### Analysis

Weights were applied to our data to align with national census benchmarks, taking into account selection probabilities (balanced by sex, age, education, race/ethnicity, and region) and non-response (Dennis, 2019). Descriptive statistics were computed for sociodemographic variables and COVID risk and protective behaviors. Bivariate analyses, including independent samples T-tests and one-way Analysis of Variance tests, were used to assess differences in mental health based on participant characteristics. A multivariable linear regression model was conducted with mental health (MHI-5) as the outcome variable. Variables were selected for inclusion in the regression based on an a priori hypothesis and findings from the literature. Statistical analyses were conducted using IBM SPSS 24.1.

## 3. Results

### Sample

Respondents included 1040 adults (after accounting for some missing data on some study variables) ages 18 – 92 (mean 47.28; SD = 17.74), of whom 51.4% were female. Most respondents identified as non-Hispanic White (62.9%) followed by Hispanic (17.4%), non-Hispanic Black (11.4%), non-Hispanic Asian (4.4%), and other or two or more races (4.4%). Sixty-one percent were employed, 34.0% had a bachelor’s degree or above, and 44.1% identified as Democrat/leaning Democrat while 37.9% identified as Republican/leaning Republican. Most respondents reported taking measures to prevent the spread of COVID as well as taking at least one action that put them at higher risk of contracting COVID. Table 1 shows the weighted sample demographics and COVID-related behaviors. The mean MHI-5 score was 67.22 (SD = 21.35) on a scale of 0 – 100, with higher scores indicating better mental health.

### Factors Associated with Mental Health: Demographics and Background

As shown in Table 1, bivariate analyses demonstrated that reported mental health differed by age (*p* < .001), biological sex (*p* < .001), income (*p* < .001), employment (*p* < .001), and political affiliation (*p* < .001). Additionally, those with a history of opioid misuse or criminal justice involvement reported poorer mental health (*p* < .001).

The multivariable linear regression model accounted for 22.5% of the variance in mental health scores (F (28, 1011) = 10.50, *p* < .001). The model demonstrated several factors associated with mental health, including gender, race, age, and political party affiliation among others. For example, those ages 65 and over (B = 18.39, 95% CI = 13.52, 23.25, *p* < .001) had higher MHI-5 than those ages 18 – 25. Respondents who indicated having a personal history of criminal justice involvement (B = −6.56, 95% CI = −10.05, −3.06, *p* < .001) or opioid misuse (B = −9.98, 95% CI = −14.74, −5.23, *p* < .001) reported significantly poorer mental health compared to those without either history. Political party was another major factor associated with mental health, as respondents identifying as Democrat reported poorer mental health than Republicans (B = −6.23, 95% CI = −9.56, −2.90, *p* < .001). See Table 2 for all regression results.

### COVID-19 Protective Behaviors Associated with Mental Health

All protective behaviors and several risk behaviors, including going to a friend or neighbor’s residence (*p* < .001), attending a gathering with more than 10 people (*p* < .001) and having close contact with others who were not wearing a mask (*p* < .001) were associated with poorer reported mental health at the bivariate level.

The regression also demonstrated that those who took any risk behavior reported better mental health compared to those who didn’t take any risk behaviors (B = 5.26, 95% CI = 2.30, 8.23, *p* = .001). All bivariate results are shown in Table 1.

Compared to those who indicated taking all four protective behaviors (68%, N = 707), those who indicated that at least 3 of the four protective behaviors weren’t necessary reported better mental health (B = 7.00, 95% CI = 1.61, 12.38, *p* = .011). Similarly, those who were taking some protective behaviors, but indicated at least one wasn’t necessary reported better mental health (B = 4.44, 95% CI = 0.529, 8.36, *p* = .026). A major predictor of poorer reported mental health was having a household member die from COVID-19 (B = −15.59, 95% CI = −25.57, −5.61, *p* = .002).

## 4. Discussion

Our findings highlight differences in mental health based on concern about COVID-19, biological sex, race, age, employment status, and political party affiliation as well as opioid and criminal justice involvement histories. These differences are important to consider as the United States continues to address the mental health needs of the population while progressing through new phases of the COVID-19 pandemic and preparing for the next big event.

Research on disaster mental health suggests there are different mental health needs at various phases for many following a disaster, such as COVID-19. Literature has shown increased symptoms of depression and anxiety and differences based on age, risk of serious illness from COVID-19, employment, and pre-existing mental health conditions (Czeisler et al., 2021; Giuntella et al., 2021; Holman et al., 2020; Shiina et al., 2020; Zhou et al., 2020). Our findings augment the literature, suggesting that mental health needs differ between sub-groups based on sociodemographic and personal characteristics.

Our most notable finding is that those who are more concerned about COVID-19 have poorer mental health. We used engagement in protective and risk behaviors as proxies for a person’s concern about COVID-19 and found that those who indicate that protective behaviors aren’t necessary or that they’ve engaged in a non-socially distanced activity report better mental health. These findings align with literature from Japan early in the pandemic that showed those with less anxiety related to COVID-19 were more likely to take risk behaviors (Holman et al., 2020; Shiina et al., 2020). Our findings may suggest that people not taking social distancing measures and less concerned about COVID-19 may not be subject to the mental health consequences of loneliness, such as depression (Killgore et al., 2020).

Research on disaster mental health has found that a person’s interpretation of trauma as a threat rather than the event itself is associated with negative psychological reactions (Ehlers & Clark, 2000; Pinto et al., 2015; Makwana, 2019). Similarly, our findings suggest that those who have not interpreted the COVID-19 pandemic as a serious threat to their health have not experienced the mental health consequences of those who are concerned about illness from COVID-19.

Our findings related to political party affiliation may reinforce this theory. We found that those who identify as Republican have better mental health than other groups. While potentially a product of election-related stress, research has demonstrated that Republicans are less worried about contracting COVID-19 (Clinton et al., 2021), and thus our findings may reinforce that the interpretation of COVID-19 as a threat influences mental health. This notion highlights the difficulty in encouraging uptake of protective behaviors during public health emergencies while not simultaneously increasing levels of anxiety about the emergency itself.

Consistent with other findings, we found younger adults reported poorer mental health compared to older adults (Czeisler et al., 2020; Zhou et al., 2020), and those who were unemployed reported poorer mental health. Interestingly, bivariate analyses showed no major differences in mental health by race, but the regression shows that Black and Hispanic participants have better reported mental health than White participants. This finding was surprising given the evidence that minorities have been disproportionately negatively impacted by COVID-19 (Gold et al., 2020) and early data suggesting higher suicide rates among racial minorities (Mitchell & Li, 2021). Generally, the literature has been mixed on mental health among different races (Zhou et al., 2020), and our findings emphasize the need to further explore mental health and race.

Our findings also shed light on the mental health of two vulnerable groups: those with a personal history of opioid misuse or criminal justice involvement. Literature has suggested that these groups may be particularly affected by COVID-19 (Ramaswamy et al., 2020; Khatri & Perrone, 2020; Schimmel & Manini, 2020). For example, studies suggest that those with an opioid use disorder had less access to treatment and harm reduction services and potentially increased substance use due to limited social connection and other activities (Schimmel & Manini, 2020; Galarneau et al., 2021). Further, the deleterious mental health effects from incarceration and the stressors of transitioning back into the community have been documented, (Sugie & Turney, 2017; Kendall et al., 2018) and recent research suggests that COVID-19 has exacerbated these challenges making them more vulnerable to these mental health implications (Ramaswamy et al., 2020). Our findings further underscore the importance of addressing mental health among these populations.

## 5. Limitations

This study had several limitations. First, we relied on the self-reported MHI-5 scale, rather than clinical diagnoses. Additionally, these data are from a cross-sectional study, so we did not have a baseline mental health score for respondents. Similarly, these data are correlational and cross-sectional, so we cannot assume causality. There are also potential confounding factors that contributed to poor mental health for which we did not control in our regression, including racial and police brutality protests, the proximate election, and pre-existing mental health conditions among participants.

## 6. Conclusion

Our findings capture the mental health of a representative national sample of adults in the United States at a unique time—late October 2020—as the compilation of the pandemic, racial protests, and the presidential election characterized this period in the United States. Specifically, our findings demonstrate that mental health differs based on sociodemographic factors and concern about the COVID-19 pandemic, highlighting potential implications for public health messaging that appropriately promotes protection while not exacerbating anxiety. Additionally, with a death toll of over 5 million globally (Centers for Disease Control and Prevention, 2021), our findings emphasize the deep mental health implications of the pandemic, since knowing someone who died related to COVID-19 was a strong predictor of mental health. Our findings point out the importance of continuing to monitor mental health throughout the pandemic, particularly as new variants, vaccine distribution, and policies evolve. Our results confirm the need for widespread mental health interventions during the COVID-19 pandemic and beyond, including increasing connection to and promotion of mental health services. Specifically, efforts should focus on the mental health needs of particular sub-populations, such as those with a higher level of concern for COVID-19 or faced exacerbated challenges already faced by those with histories of opioid use disorder and criminal justice involvement. Drawing from disaster mental health literature, (Makwana, 2019; North et al., 2021) we can anticipate that the mental health of the population will continue to change over the next several years and continue to offer mental health support to communities will be essential.

## Figures and Tables

**Table 1.** Sample demographics frequencies and group disfferences in MHI-5 means (n = 1040)^a^.

Characteristic	N (%)	MHI-5 Mean (SD)	95% CI	*p*-value	
Age				<.001	
18 – 25	137 (13.2)	59.68 (18.41)	56.57, 62.79		
26 – 39	263 (25.3)	62.40 (21.36)	59.81, 65.00		
40 – 54	235 (22.6)	64.47 (23.29)	61.48, 67.46		
55 – 64	192 (18.5)	71.04 (20.44)	68.13, 73.95		
65+	213 (20.5)	77.62 (16.77)	75.36, 79.89		
Sex				.001	
Male	506 (48.6)	69.48 (21.10)	67.64, 71.32		
Female	535 (51.4)	65.09 (21.39)	63.27, 66.90		
Race				0.182	
Black	118 (11.4)	67.49 (22.32)	63.42, 71.55		
White	654 (62.9)	67.42 (21.57)	65.77, 69.08		
Other/2+	46 (4.4)	67.68 (21.17)	61.37, 73.99		
Hispanic	177 (17.0)	68.14 (21.02)	65.02, 71.26		
Asian	45 (4.4)	59.59 (15.60)	54.92, 64.25		
Income				<.001	
less than $25,000	249 (24.0)	61.92 (22.12)	59.16, 64.67		
$25 - $49,000	234 (22.5)	67.38 (21.21)	64.66, 70.11		
$50 - $84,000	248 (23.8)	68.33 (22.62)	65.50, 71.15		
$85 - $150,000	240 (23.1)	70.46 (19.22)	68.02, 72.91		
over $150,000	68 (6.5)	70.63 (17.97)	66.28, 74.97		
Employment				<.001	
Employed	635 (61.0)	66.44 (21.84)	64.74, 68.14		
Unemployed, looking for work	53 (5.1)	57.92 (19.39)	52.56, 63.29		
Unemployed, retired/disabled/other	353 (33.9)	70.02 (20.25)	67.90, 72.14		
Education				0.082	
Less than high school	102 (9.8)	62.74 (19.73)	58.87, 66.61		
High school or some college	584 (56.2)	67.73 (21.62)	65.98, 69.49		
Bachelor’s degree or above	354 (34.0)	67.67 (21.25)	65.45, 69.89		
Political Affiliation				<.001	
Democrat	349 (33.5)	64.28 (20.94)	62.08, 66.49		
Lean Democrat	110 (10.5)	64.47 (20.46)	60.60, 68.34		
Don’t Lean/Independent/None	187 (18.0)	66.06 (22.11)	62.88, 69.25		
Lean Republican	77 (7.4)	62.76 (23.55)	57.40, 68.12		
Republican	318 (30.5)	73.16 (19.92)	70.96, 75.36		
Personal Opioid History				<.001	
Yes	79 (7.6)	53.36 (19.52)	48.97, 57.74		
No	962 (92.4)	68.36 (21.10)	67.02, 69.69		
Personal Criminal Justice History				<.001	
Yes	177 (17.0)	60.35 (23.21)	56.91, 63.80		
No	864 (83.0)	68.63 (20.68)	67.25, 70.01		
Household COVID Death				<.001	
Yes	16 (1.5)	43.40 (11.53)	37.26, 49.53		
No	1024 (98.5)	67.60 (21.26)	37.26, 49.53		
Washing hands more than usual				<.001	
No, I don’t think it is necessary	69 (6.7)	71.06 (22.11)[]	65.76, 73.37		
No, I would like to but I cannot	47 (4.5)	54.74 (16.93)[]	49.76, 59.72		
Yes	924 (88.8)	67.57 (21.30)[]	66.19, 68.94		
Limiting interactions to groups of 10 or less	less			<.001	
No, I don’t think it is necessary	154 (14.8)	74.99 (19.18)	71.94, 78.04		
No, I would like to but I cannot	93 (8.9)	59.30 (20.23)	55.13, 63.46		
Yes	793 (76.2)	66.64 (21.44)	65.14, 68.13		
Keep a 6-foot distance from others				.008	
No, I don’t think it is necessary	89 (8.6)	73.26 (19.73)	69.11, 77.42		
No, I would like to but I cannot	78 (7.5)	63.44 (20.95)	58.72, 68.16		
Yes	873 (83.9)	66.94 (21.45)	65.52, 68.37		
Wearing a mask when leaving home				<.001	
No, I don’t think it is necessary	96 (9.2)	73.77 (20.55)	69.60, 77.94		
No, I would like to but I cannot	35 (3.3)	56.13 (15.28)	50.86, 61.40		
Yes	910 (87.5)	66.96 (21.43)	65.56, 68.35		
Gone to a Restaurant or Bar				.794	
No	639 (61.5)	67.08 (21.58)	65.41, 68.76		
Yes	401 (38.5)	67.44 (20.99)	65.38, 69.50		
Gone to a friend/neighbor’s residence				.001	
No	415 (39.9)	64.43 (22.70)	62.25, 66.62		
Yes	625 (60.1)	69.07 (20.21)	67.49, 70.66		
Attended a gathering with 10+ people				.001	
No	762 (73.2)	65.92 (21.72)	64.38, 67.47		
Yes	279 (26.8)	70.77 (19.90)	68.43, 73.12		
Shared items with non-household members				.337	
No	914 (87.8)	67.01 (21.71)	65.61, 68.42		
Yes	127 (12.2)	68.74 (18.53)	65.48, 72.00		
Close contact with others who were not wearing masks				.028
No	564 (54.2)	65.90 (22.54)	64.04, 67.76	
Yes	476 (45.8)	68.79 (19.76)	67.01, 70.57	
Close contact with others when you were not wearing a mask				.189
No	633 (60.9)	66.54 (22.13)	64.81, 68.27	
Yes	407 (39.1)	68.28 (20.05)	66.33, 70.24	

a Data weighted to national census benchmarks for sex, age, education, race/ethnicity, region.

b *p*-value determined by one-way Analysis of Variance test or independent samples t-test, with *p* < .05 considered significant.

**Table 2.** Multivariable linear regression of MHI-5 scale scores (n = 1040)^a^.

Characteristic	B (95%CI^b^)	*p*-value^c^
Protective behaviors		
Take all protective behaviors	Reference	-
Believe most protective behaviors are unnecessary	7.00 (1.61, 12.38)	.011
Take 2 – 3 protective behaviors, others not necessary	4.45 (0.53, 8.36)	.026
Would like to, but cannot take protective behavior	−.30 (−3.90, 3.31)	.872
Have taken at least one risk behavior		
No	Reference	-
Yes	5.26 (2.30, 8.23)	.001
Sex		
Female	Reference	-
Male	5.07 (2.63, 7.52)	<.001
Race		
White	Reference	-
Black	6.28 (2.26, 10.30)	.002
Other/2+	7.44 (1.34, 13.54)	.017
Hispanic	5.16 (1.83, 8.49)	.002
Asian	−1.20 (−7.38, 4.97)	.703
Household Income		
<$25,000	Reference	-
$25 - $49,000	4.86 (1.33, 8.39)	.007
$50 - $84,000	3.35 (−.30, 6.99)	.072
$85 - $150,000	5.36 (1.54, 9.19)	.006
$150,00+	4.35 (−1.33, 10.02)	.133
Age		
18 – 25	Reference	-
26 – 39	3.38 (−.91, 7.68)	.122
40 – 54	4.46 (−.05, 8.96)	.052
55 – 64	11.22 (6.54, 15.90)	<.001
65+	18.39 (13.52, 23.25)	<.001
Political Party		
Republican	Reference	-
Lean Republican	−9.40 (−14.20, −4.59)	<.001
No lean/independent	−2.94 (−6.66, 0.79)	.123
Lean Democrat	−6.73 (−11.14, −2.32)	.003
Democrat	−6.23 (−9.56, −2.90)	<.001
Personal criminal justice history		
No	Reference	-
Yes	−6.56 (−10.05, −3.06)	<.001
Personal history of ever misusing opioids		
No	Reference	-
Yes	−9.98 (−14.74, −5.23)	<.001
Household member died from COVID		
No	Reference	-
Yes	−15.59 (−25.57, −5.61)	<.002
Employment Status		
Employed	Reference	-
On temporary layoff or looking for work	−5.77 (−11.37, −.17)	.043
Unemployed, retired/disabled/other	−1.83 (−4.80, 1.14)	.227
Education Level		
BA or above	Reference	-
HS diploma or some college/associates degree	1.46 (−1.40, 4.33)	.316
Less than high school	−0.06 (−4.99, 4.87)	.981

Unadjusted R^2^ = .22, *p* < .001.

a Data weighted to national census benchmarks for sex, age, education, race/ethnicity, region.

b CI = confidence interval.

c *p* < .05 considered significant.
